# Caffeine ingestion stimulates plasma carnitine clearance in humans

**DOI:** 10.14814/phy2.15615

**Published:** 2023-02-17

**Authors:** Benjamin T. Wall, David Machin, Mandy V. Dunlop, Francis B. Stephens

**Affiliations:** ^1^ Department of Public Health and Sport Sciences University of Exeter Exeter UK

**Keywords:** caffeine, carnitine, OCTN2

## Abstract

Increasing skeletal muscle carnitine content can manipulate fuel metabolism and improve exercise performance. Intravenous insulin infusion during hypercarnitinemia increases plasma carnitine clearance and Na^+^‐dependent muscle carnitine accretion, likely via stimulating Na^+^/K^+^ ATPase pump activity. We hypothesized that the ingestion of high‐dose caffeine, also known to stimulate Na^+^/K^+^ ATPase activity, would stimulate plasma carnitine clearance during hypercarnitinemia in humans. In a randomized placebo‐controlled study, six healthy young adults (aged 24 ± 5 years, height 175 ± 8 cm, and weight 70 ± 13 kg) underwent three 5‐h laboratory visits involving the primed continuous intravenous infusion of l‐carnitine (CARN and CARN + CAFF) or saline (CAFF) in parallel with ingestion of caffeine (CARN + CAFF and CAFF) or placebo (CARN) at 0, 2, 3, and 4 h. Regular blood samples were collected to determine concentrations of blood Na^+^ and K^+^, and plasma carnitine and caffeine, concentrations. Caffeine ingestion (i.e., CAFF and CARN + CAFF conditions) and l‐carnitine infusion (i.e., CARN and CARN + CAFF) elevated steady‐state plasma caffeine (to ~7 μg·mL^−1^) and carnitine (to ~400 μmol·L^−1^) concentrations, respectively, throughout the 5 h infusions. Plasma carnitine concentration was ~15% lower in CARN + CAFF compared with CARN during the final 90 min of the infusion (at 210 min, 356 ± 96 vs. 412 ± 94 μmol·L^−1^; *p* = 0.0080: at 240 min, 350 ± 91 vs. 406 ± 102 μmol·L^−1^; *p* = 0.0079: and at 300 min, 357 ± 91 vs. 413 ± 110 μmol·L^−1^; *p* = 0.0073, respectively). Blood Na^+^ concentrations were greater in CAFF and CARN + CAFF compared with CARN. Ingestion of high‐dose caffeine stimulates plasma carnitine clearance during hypercarnitinemia, likely via increased Na^+^/K^+^ ATPase activity. Carnitine co‐ingestion with caffeine may represent a novel muscle carnitine loading strategy in humans, and therefore manipulate skeletal muscle fuel metabolism and improve exercise performance.

## 1 INTRODUCTION

Carnitine primarily exists within skeletal muscle where it is a crucial cofactor for the rate limiting step of fat oxidation and as a buffer to maintain oxidative flux through the pyruvate dehydrogenase complex (PDC) at times of high glycolytic demand (e.g., high intensity exercise) (Stephens, Constantin‐Teodosiu, & Greenhaff, 2007). Accordingly, carnitine has long been proposed as an attractive ergogenic aid to improve fat oxidation therapeutically (e.g., in patients with type‐2 diabetes) or accelerate fat and/or oxidative glucose utilization to improve exercise performance (Stephens, 2018).

Intravenous (iv; Stephens et al., 2006a) or oral (Wächter et al., 2002) carnitine administration per se does not increase muscle carnitine uptake or muscle storage and consequently, fails to modulate muscle fuel metabolism or exercise performance. This is attributable to sodium (Na^+^)‐dependent muscle carnitine transport being saturated at normal plasma concentrations (Tamai et al., 1998). However, intravenous insulin infusion during hypercarnitinemia results in significant plasma carnitine clearance into skeletal muscle, likely via stimulating Na^+^/K^+^ ATPase pump activity (Stephens et al., 2006a, 2006b; Stephens, Constantin‐Teodosiu, Laithwaite, et al., 2007). Muscle carnitine loading is also achievable by nutritional means; daily co‐ingestion of carbohydrate (CHO; to stimulate insulin secretion) increases muscle carnitine uptake and thus whole‐body carnitine retention compared with carnitine ingestion alone (Shannon et al., 2016; Stephens, Evans, Constantin‐Teodosiu, & Greenhaff, 2007). Resultantly, 12–24 weeks CHO (or mixed macronutrient) and carnitine co‐ingestion increases muscle carnitine storage by ~10%–20% (Chee et al., 2021; Shannon et al., 2016; Stephens et al., 2013; Wall et al., 2011).

Evidence suggests that caffeine also stimulates Na^+^/K^+^ ATPase pump activity in rodent (Rogus et al., 1977) and human (Lindinger et al., 1996) skeletal muscles. The aim of the present study was to test the hypothesis that the ingestion of high‐dose caffeine would stimulate plasma carnitine clearance during hypercarnitinemia in healthy humans.

## 2 METHODS

### 2.1 Ethics statement

The study was approved by the Department of Sport and Health Sciences, University of Exeter's Research Ethics Committee (reference: 180207/B/01) in accordance with the latest Declaration of Helsinki (version October 2013). All participants were informed on the nature and risks of the experiment before written informed consent was obtained.

### 2.2 Participants

Six healthy, young, omnivorous adults (4 males and 2 females, age 24 ± 2 years, body mass 68 ± 5 kg, height 173 ± 4 cm, BMI 23 ± 1 kg/m^2^) were included in the present study. Prior to inclusion, participants attended the laboratory for a routine medical screening to ensure their eligibility to take part.

### 2.3 Experimental protocol

In a randomized, placebo‐controlled, double‐blind (for both participant and experimenter and for both caffeine and carnitine administrations) trial, participants arrived at the laboratory on three occasions at 11:00 having followed a controlled diet the evening prior (900 kcals; 20/25/55% fat/protein/CHO, respectively) and for breakfast (600 kcals; 20/25/55% fat/protein/CHO, respectively), with all food provided by the research team for consumption at home with appropriate instruction. These laboratory visits were to conduct three randomly assigned, counterbalanced (order) experimental test days: (1) carnitine only (CARN); (2) caffeine only (CAFF); (3) carnitine + caffeine (CARN + CAFF). Participants rested in a semi‐supine position on a bed for the 5 h duration of each experimental test day. Prior to the start of the experiment, two cannulas were placed; (1) retrogradely into a dorsal hand vein of the non‐dominant hand for arterialized venous blood sampling (Gallen & Macdonald, 1990), and (2) into an antecubital vein of the contralateral arm for the infusions of saline or l‐carnitine (Carnitor levocarnitine; Leadiant BioSciences Inc. Gaaithersburg, MD). At *t* = 0 min, following a baseline blood sample collection, participants ingested 6 mg/kg body mass (bm) of caffeine (Caffeine powder ReagentPlus®; Merck Life Science UK Limited) (CAFF and CARN + CAFF visits) or an equivalent dose of a dextrose placebo (CARN visit) made up into capsule form. A bolus of 15 mg/kg bm l‐carnitine (CARN and CARN + CAFF visits) or the equivalent volume of 0.9% saline (CAFF visit) was then intravenously infused over 10 min. Thereafter, the infusions were reduced to 10 mg/kg bm/h l‐carnitine (or equivalent volume of saline) for the following 290 min. At 2, 3, and 4 h, participants ingested additional 1 mg/kg bm doses of caffeine or dextrose placebo (making a total dose of 9 mg/kg bm or 613 ± 45 mg over each test day). Further arterialized venous blood samples were collected every 30 min over the duration of the infusions. Participants consumed a 50 mL bolus of water with each capsule (i.e., total volume ingested of 200 mL at controlled intervals), but were not permitted any further water throughout the duration of the infusion period. The dose of caffeine was selected as a “high dose,” previously been shown to be well tolerated (Spriet, 1992) and fed in multiple doses to maintain persistently high circulating caffeine concentrations during the entire period of hypercarnitinemia.

### 2.4 Sample analyses

Arterialized venous blood samples were immediately analyzed for blood glucose and lactate (YSI 2300 STATplus, YellowSprings Instruments), and Na^+^ and K^+^ (pHOx Ultra, NOVA Biomedical) concentrations, and were subsequently collected in BD Vacutainer® PST Lithium Heparin tubes and centrifuged at 2900*g* at 4°C for 10 min. Blood plasma was then recovered and immediately frozen in liquid nitrogen and was subsequently stored at −80°C. Plasma caffeine and free carnitine concentrations were assayed at a later date via high‐performance liquid chromatography (Holland et al., 1998) and a radioenzymatic assay (Cederblad et al., 1982), respectively. The pHOx Ultra analyzer did not work for one of the participants and so blood Na^+^ and K^+^ concentration data are presented as *n* = 5.

### 2.5 Statistics

All data are expressed as mean ± SD. Data were analyzed using repeated measures two‐way ANOVAs (time *x* treatment, with treatment representing the repeated measure). In case of a significant interaction, Bonferroni post hoc tests were applied to locate individual differences. Violations of normality were tested for using the Shapiro–Wilk test, and no considerable violations were found (*p* > 0.05). All statistics were carried out on the statistical package GraphPad Prism (version 8.4.2) and statistical significance was set at *p* < 0.05.

## 3 RESULTS

### 3.1 Plasma variables

**FIGURE 1:**
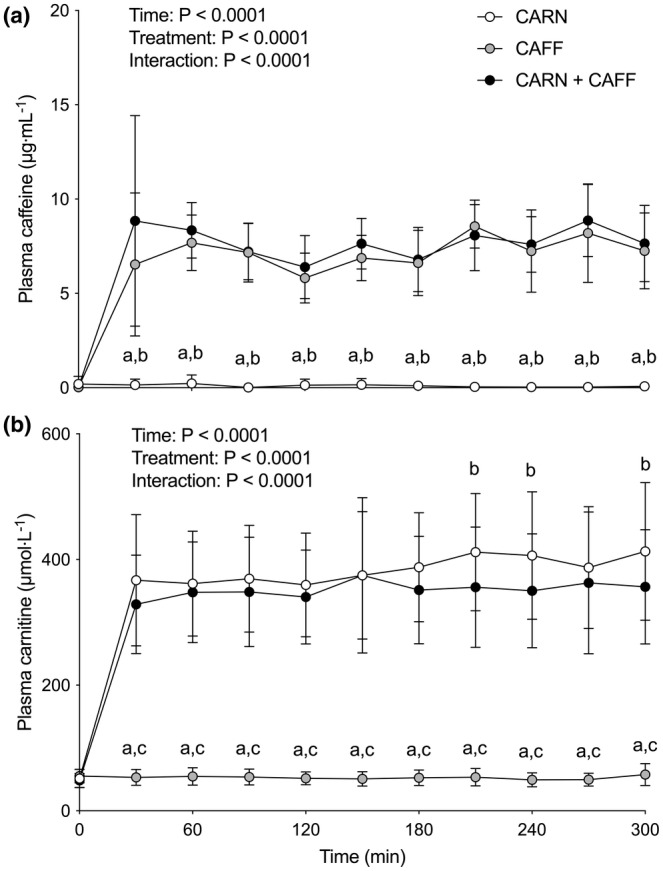
Plasma caffeine (a) and free carnitine (b) concentrations at baseline (i.e., 0 min) and throughout 5 h primed and continuous intravenous infusions of saline (CAFF) or l‐carnitine (CARN and CARN + CAFF), and ingestion of placebo (CARN) or 9 mg/kg body mass (bm) caffeine (consumed as 6 mg/kg bm at baseline and 1 mg/kg bm after 120, 180, and 240 min) (CAFF and CARN + CAFF) administered in a randomized, counterbalanced, crossover, double‐blind fashion in six healthy young adults. Data (*n* = 6) are presented as means ± SD and analyzed with repeated measures two‐way ANOVAs (time and treatment; main effects depicted) with Bonferroni post hoc tests where “*a*” denotes a significant difference between corresponding values of CARN and CAFF (*p* < 0.0001), “*b*” denotes a significant difference (*p* < 0.0001 in a and *p* < 0.01 in b) between corresponding values of CARN and CARN + CAFF, and “*c*” denotes a significant difference (*p* < 0.0001) between corresponding values of CAFF and CARN + CAFF.

Plasma caffeine (a) and carnitine (b) concentrations are displayed in Figure 1. From equivalent baseline values (0.19 ± 0.41, 0.03 ± 0.02, and 0.03 ± 0.02 μg·mL^−1^ in CARN, CAFF, and CARN + CAFF conditions, respectively), plasma caffeine concentrations increased immediately (by 30 min) to 6.54 ± 3.79 and 8.84 ± 5.58 μg·mL^−1^ following supplement ingestion in the CAFF and CARN + CAFF conditions, but remained unchanged throughout the experiment in CARN (effects of time, treatment, and time × treatment interaction; all *p* < 0.0001). Plasma caffeine concentrations remained elevated in CAFF and CARN + CAFF (compared with baseline and CARN at every time point; all *p* < 0.0001) throughout the experiment, with no differences between CAFF and CARN + CAFF conditions.

Plasma carnitine concentrations were also comparable across conditions at baseline (52 ± 14, 55 ± 11, and 48 ± 11 μmol·L^−1^ in CARN, CAFF, and CARN + CAFF conditions, respectively). The initiation of intravenous infusions rapidly (by 30 min) increased plasma carnitine concentrations (~7‐fold) in CARN and CARN + CAFF, but not CAFF (effects of time, treatment, and time × treatment interaction; all *p* < 0.0001). Although plasma carnitine concentrations remained elevated in CARN and CARN + CAFF throughout the entire experiment compared with baseline and CAFF (all *p* < 0.0001), for the final 2 h of the experiment values were lower in CARN + CAFF compared with CAFF conditions (at 210 min, 356 ± 96 vs. 412 ± 94 μmol·L^−1^; *p* = 0.0080: at 240 min, 350 ± 91 vs. 406 ± 102 μmol·L^−1^; *p* = 0.0079: and at 300 min, 357 ± 91 vs. 413 ± 110 μmol·L^−1^; *p* = 0.0073, respectively).

### 3.2 Blood variables

**FIGURE 2:**
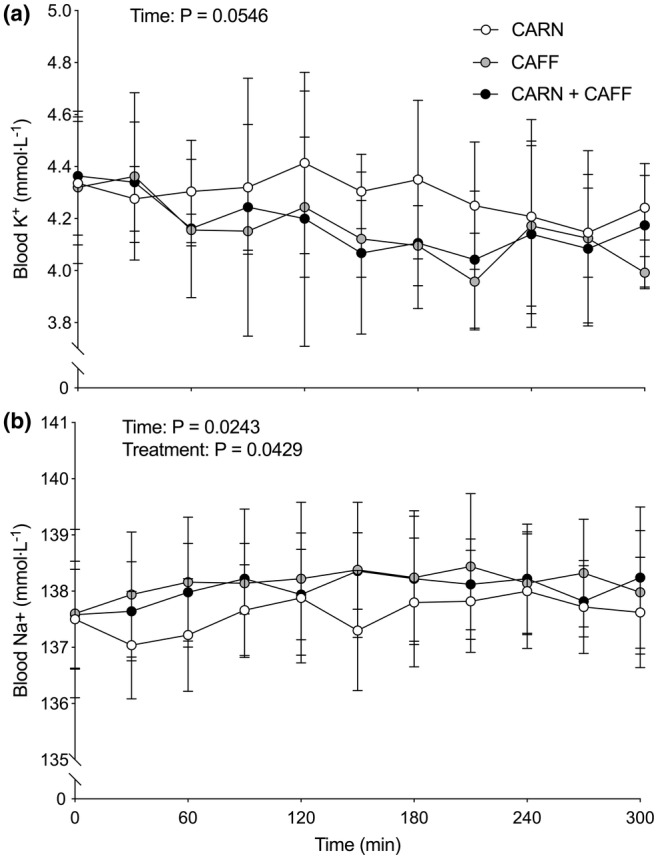
Blood potassium (K^+^; a) and sodium (Na^+^; b) concentrations at baseline (i.e., 0 min) and throughout 5 h primed and continuous intravenous infusions of saline (CAFF) or l‐carnitine (CARN and CARN + CAFF), and ingestion of placebo (CARN) or 9 mg/kg body mass (bm) caffeine (consumed as 6 mg/kg bm at baseline and 1 mg/kg bm after 120, 180, and 240 min) (CAFF and CARN + CAFF) administered in a randomized, counterbalanced, crossover, double‐blind fashion in six healthy young adults. Data (*n* = 5) are presented as means ± SD and analyzed with repeated measures two‐way ANOVAs (time and treatment; main effects depicted) with Bonferonni post hoc tests.

**FIGURE 3:**
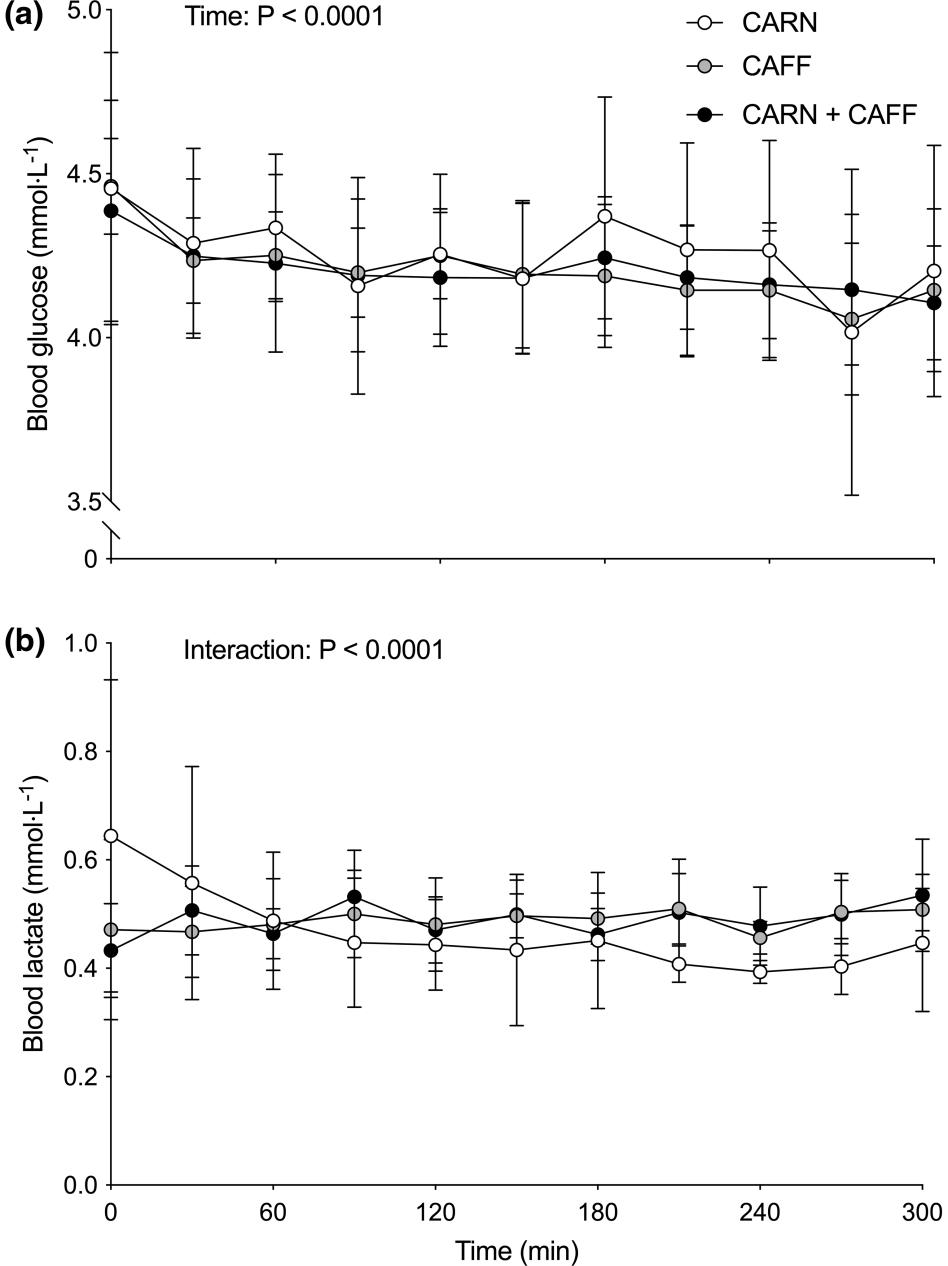
Blood glucose (a) and lactate (b) concentrations at baseline (i.e., 0 min) and throughout 5 h primed and continuous intravenous infusions of saline (CAFF) or l‐carnitine (CARN and CARN + CAFF), and ingestion of placebo (CARN) or 9 mg/kg body mass (bm) caffeine (consumed as 6 mg/kg bm at baseline and 1 mg/kg bm after 120, 180, and 240 min) (CAFF and CARN + CAFF) administered in a randomized, counterbalanced, crossover, double‐ blind fashion in six healthy young adults. Data (*n* = 6) are presented as means ± SD and analyzed with repeated measures two‐way ANOVAs (time and treatment; main effects depicted).

Blood K^+^ (a) and Na^+^ (b) concentrations, and blood glucose (a) and lactate (b) concentrations are shown in Figures 2 and 3, respectively. Blood K^+^ concentrations tended to decrease over time (*p* = 0.055) and equivalently so in all conditions (treatment *p* = 0.4720; time × treatment interaction *p* = 0.8399). Conversely, blood Na^+^ concentrations increased over time (*p* = 0.0243) with a treatment effect evident (*p* = 0.0429) such that blood Na^+^ concentrations were higher in CAFF compared with the CARN condition (*p* = 0.0451). Blood glucose concentrations decreased over time (*p* < 0.0001) comparably across conditions (treatment effect, *p* = 0.8270; time × treatment interaction, *p* = 0.5501). No time (*p* = 0.4556) or treatment (*p* = 0.7746) effects were observed for blood lactate concentration but a significant interaction was detected (*p* < 0.0001) such that lactate concentration appeared to be lower in CARN compared with CAFF and CARN + CAFF over the final 90 min of infusion.

## 4 DISCUSSION

In the present study, we reasoned that caffeine ingestion would stimulate Na^+^‐dependent muscle carnitine uptake secondary to its reported effects of increasing Na^+^/K^+^ ATPase pump activity (Lindinger et al., 1996; Rogus et al., 1977). We used the ingestion of a total dose of caffeine of 9 mg/kg body mass (bm) administered as a large bolus at the outset (6 mg/kg bm) and in three smaller (1 mg/kg bm) consecutive boluses after 2, 3, and 4 h of the experiment. This approach resulted in an immediate and considerable rise in plasma caffeine concentrations, which was sustained in a relatively steady‐state manner throughout the experiment (see Figure 1a). In parallel, we created steady‐state and supraphysiological hypercarnitineemia consistent with our previous work (e.g., Stephens et al., 2006a) via the primed and continuous intravenous infusions of carnitine (see Figure 1b). This combination of high circulating levels of caffeine and carnitine resulted in increased plasma carnitine clearance (i.e., 10%–15% lower plasma carnitine concentration when high caffeine concentrations are present) for the final hour of experimentation. Though we did not confirm that urinary carnitine elimination was comparable across conditions, we know that renal reabsorption of carnitine is saturated during intravenous carnitine infusion (Stephens et al., 2006a; Stephens, Constantin‐Teodosiu, Laithwaite, et al., 2007), and that carnitine is primarily (>95%) stored in skeletal muscle (Stephens, Constantin‐Teodosiu, & Greenhaff, 2007), suggesting that the increased plasma carnitine clearance was a result of increased Na^+^‐dependent muscle carnitine uptake. Our data support this thesis, given we observed a (visual) decrease over time in blood K^+^ concentrations, a statistical increase in blood Na^+^ concentrations, and a higher blood lactate concentration in the two caffeine conditions (see Figure 2 and Figure 3), which is in line with what is typically seen with caffeine ingestion altering blood Na^+^/K^+^ balance and metabolism (Lindinger et al., 1996).

We have previously demonstrated that hypercarnitinemia combined with hyperinsulinemia (achieved via intravenous infusion of insulin [105 mU·m^−2^·min^−1^] for 6 h) also increased plasma carnitine clearance by ∼15% and resulted in an increase in skeletal muscle total carnitine content of ∼15% (Stephens et al., 2006a). This finding was also in agreement with the hypothesis that increasing sarcolemmal Na^+^/K^+^‐ATPase pump activity would increase Na^+^–carnitine co‐transport via OCTN2, given that insulin increases Na^+^/K^+^‐ATPase activity by increasing translocation of α2 and β1 pump subunits from an intracellular storage site to the plasma membrane (Sweeney & Klip, 1998), and the sensitivity of the Na^+^/K^+^‐ATPase to intracellular Na^+^ (Clausen, 1986). Indeed, muscle carnitine transport is inhibited by the potent Na^+^ /K^+^‐ATPase inhibitor ouabain (Georges et al., 2000; Rebouche, 1977). Thus, high‐dose caffeine ingestion appears to be as potent as high circulating insulin concentrations at stimulating plasma carnitine clearance into skeletal muscle. It should be noted, however, that caffeine has also been shown to *impair* carnitine uptake into C2C12 cells (Shaw et al., 2017). The reason(s) for lack of congruence between these in vitro data and our human work (both previous and the present study) is not obvious but could be linked to differing Na^+^ and/or carnitine gradients achieved under in vivo conditions and those with in vitro pharmacological manipulations known to modulate Na^+^/K^+^‐ATPase pump activity. Moreover, the in vitro work also did not confirm the stimulatory role of insulin in promoting muscle carnitine uptake (Shaw et al., 2017), likely because cellular carnitine uptake was already maximal under non‐stimulated conditions.

While the plasma carnitine concentration achieved in the present study is around fivefold higher than that achieved with oral carnitine ingestion, we know that other interventions that increase Na^+^/K^+^ ATPase pump activity can increase muscle carnitine uptake to a similar degree following oral L‐carnitine administration (Shannon et al., 2016). Taken together with what we know about the prolonged co‐ingestion of carnitine with insulinogenic macronutrients, our current data suggest that daily caffeine and carnitine co‐ingestion represents a promising longer‐term non‐caloric nutritional strategy to practically and feasibly induce muscle carnitine loading in humans and, therefore, manipulate muscle fuel metabolism and exercise performance.

## AUTHOR CONTRIBUTIONS

Benjamin T. Wall, David Machin, and Francis B. Stephens contributed to the conception and design of the experiment. Benjamin T. Wall, David Machin, Mandy V. Dunlop, and Francis B. Stephens contributed to data collection and analysis. Benjamin T. Wall, David Machin, and Francis B. Stephens drafted the manuscript. All authors contributed to the interpretation of the data and revision toward important intellectual content. All authors approved the final version of the manuscripts ensuring accuracy and questions related to the integrity of the research were scrutinized and resolved. All persons designated as authors qualify for authorship, and all those who qualify for authorship are listed.

## FUNDING INFORMATION

This study was funded by the University of Exeter.

## CONFLICT OF INTEREST STATEMENT

None of the authors disclose any conflicts of interest.
